# Book Reviews

**Published:** 1811-03

**Authors:** 


### CRITICAL ANALYSIS

OF

### RECENT PUBLICATIONS

IN THE

DIFFERENT BRANCHES OF PHYSIC, SURGERY, AND MEDICAL PHILOSOPHY,

### *Stock's Life of Beddoes.*

(Continued from p. 175.)

In 1790, Dr. Beddoes published at the Clarendon press, an analytical account of the writings of Mayow; under the title of Chemical Experiments and Opinions extracted from a work published in the last century.

" Till the appearance of this treatise, (observes Dr. Stock) these brilliant discoveries which had outstripped, by upwards of a century, the more tardy advances of other chemical philosophers, had either remained totally unknown, or had received only a scanty or derogatory notice."

Amidst his other pursuits, Dr. B. cultivated the science of mineralogy with great assiduity and success; and in 1791, read a paper before the Royal Society, entitled, Observations on the Affinity between Basaltes and Granite, in which he aspired to elucidate some of the most intricate questions relative to geology. In the same year he also contributed a valuable paper, containing an account of some appearances attending the conversion of cast into malleable iron. The following year he diversified his studies by writing botanical dialogues.

About this period the memorable revolution which was effecting in France, excited deep interest in every reflecting mind.

"At such a period, it was not an easy task for any person possessed of an independent mind and ardent feelings, to avoid taking a part, and expressing an opinion, upon the important questions which were daily agitating. Dr. Beddoes, at least, was not that man. He hailed the dawn of French freedom with enthusiasm, and cherished, 'through many a dark and stormy year' hopes of an issue in favour of mankind." P. 45.

In this year he wrote a treatise upon the subject of early instruction; and composed a poem, called "Alexander's Expedition to the Indian Ocean."

"This originated," he observes, "in a stratagem, which, if not entirely innocent, can be charged only with the guilt of presumption. In order to impose upon a few of their common acquaintance, the writer, in a few passages at least, attempted to assume the style of the most elegant of modern poets." (Dr. Darwin.)

In this he succeeded, but never ventured to publish the whole of the poem; part of it was inserted in the Annual Anthology in 1796. From the specimens which Dr. Stock has quoted, it seems to possess considerable merit; and many passages bear a striking resemblance to some parts of the "Botanic Garden."

Soon afterwards, he resigned his lectureship at Oxford; and about the same time, published "Observations on the Nature of Demonstrative Evidence, &c. with Reflections on Language." And a medical work, entitled, "Observations on the Nature and Cure of Calculus, Sea-scurvy, Consumption, Catarrh, and Fever; together with Conjectures upon several other Objects of Physiology and Pathology." In the first division of the work, he speaks of the great efficacy of alkaline alkaline remedies in calculus, and especially recommends the carbonate of soda,

"Of which the water of chrystallization has been dissipated by exposure to a warm dry air, or by being spread before a fire. This is combined with rather more than an equal weight of soap, and made into pills. One or two samples of the powder are taken in the course of the day." P. 77.

Immediately upon quitting Oxford, Dr. B. returned to Shiffnal, and having remained there a short time, paid a visit to Ketley. During his residence at this place, he published his celebrated history of Isaac Jenkins; an admirable moral fiction, which gives an account of the Reformation of a drunken Labourer, and his return to Habits of Sobriety and Industry. The success of this little work was extraordinary. Forty thousand copies were disposed of before the end of the year 1796; and the author issued a large impression, a few years since, at the particular request of a society for the promotion of knowledge and virtue by the distribution of books. "It was during this visit also, that he first developed those ideas on the medical use of the permanently elastic fluids, which afterwards attracted such general attention."

A pneumatic establishment was determined upon, and Dr. Beddoes, with three friends, subscribed each 200l. to institute it, Dr. B. engaging to superintend the undertaking; he also voluntarily proposed that the whole of the fees he might receive pending the issue of the experiment, should be devoted to the same purpose.

After encountering many difficulties in the prosecution of his scheme, he succeeded by the friendly agency of Mr. Edgeworth, who happened to be at Clifton at the time, in securing an establishment at the Hot Wells. Our biographer informs us,

"The society of this gentleman (Mr. Edgeworth) was a most valuable acquisition to Dr. Beddoes, upon his arrival in a place where he was personally known to so few. There existed between them a similarity of opinion upon a variety of subjects, which rendered their intercourse more pleasant and unrestrained. The science of education, in particular, had already occupied the attention of both, and the practical and enlightened views of the one were still farther extended by the philosophical theories of the other." P. 93.

Dr. Beddoes soon became more intimately connected with this excellent man by marrying one of his daughters. In 1793, he published a letter to Dr. Darwin, containing a farther explanation of his theory of the treatment of consumption, in which he points out two principle varieties of the disease. The one, he terms florid consumption, is denoted by a vivid redness of the cheeks at the accession of a paroxysm, roxysm, and an extraordinary permanent redness of the lips, tongue, and fauces.

"The eyes too, in such cases, are remarkable for their vivacity. The blood discharged by epistaxis, or hæmoptysis, has a colour evidently more fluid than usual."

From these symptoms of hyperoxygenation, he draws the following inferences.

"It is an evident consequence of my leading opinion, that a phthisical patient would take a longer time than another in being drowned, or in being suffocated in most of those airs that are unfit for respiration. Being already provided, as I suppose them to be, with a larger proportion of that principle which respiration introduces into the body, they must be able to continue, for a longer time, without the necessity of a new supply; and as the left cavities of the heart seem to be more irritable in such patients, it is probable they would be more easily recoverable from accidents of this kind, than persons in an ordinary state of health."

His attention to the effects of different gases in consumption procured him the correspondence of many eminent physicians, who were gratified with his discoveries, and disposed to assist his inquiries. He was now occupied with considering the means of establishing a hospital for the administration of pneumatic remedies; the late accomplished Duchess of Devonshire, who had been much pleased with his apparatus in Hope-square, entered into the plan with great eagerness.

"In the mean time, however, he found leisure to publish a little tract, addressed to the more humble classes of society, entitled a Guide for Self Preservation and Parental Affection. In this work, he points out in the most simple language, a variety of errors generally prevalent upon subjects in which health is concerned."

This little tract had a rapid and extensive sale. Dr. Darwin in one of his letters to the author, says of it, "I have read a little work of yours about preserving health with great pleasure. You deserve a Civic crown for saving the lives of your fellow citizens."

Soon after his marriage with Miss Edgeworth, he engaged in the superintendance of a new edition of the works of the celebrated John Brown, for the benefit of his widow and children. His motives for undertaking this task were very honourable to him; we state them in his own words, "to administer this consolation to men of genius languishing in penury and neglect; that when they are gone from the scene, some notice will be taken, for their sakes, of those nearest and dearest to them."

In this year also, he published, under the title of a "Proposal for the Improvement of Medicine," his plan for determining the power of factitious airs, by the formation of an institution institution for that specific purpose. He had previously secured the powerful assistance of Mr. Watt, to construct his apparatus. In four months a second edition with additions and improvements was published: in this, Dr. Beddoes has related many interesting experiments, illustrative of the effects of the different gasses.

"In the mean time, his attention was not unawakened by the political occurrences which took place at the close of this year. He wrote for a certain society, an address to Thomas Hardy upon his acquittal. It is conceived in a style of forcible and indignant eloquence; and animadverts with the most poignant severity upon the conduct of Mr. Pitt, in persecuting the most consistent adherents of a cause, of which, he himself had once been one of the most distinguished advocates." P. 106.

His published writings this year finished with a narrative of the good effects of opium in counteracting an excessive dose of digitalis; this appeared in the 5th vol. of the Medical Facts and Observations.

In the year 1795, his well-known edition of Brown's Elements of Medicine was given to the public. This was almost immediately followed by a translation, from the Spanish, of Gimbernat's New Method of operating in Femoral Hernia. His labours in promoting the pneumatic medicine were ardently continued, and many cases in which it had been practised, being communicated to him, he was enabled in the autumn to publish a third part of the Considerations.

"He complains, in a letter written in November of this year, that his literary pursuits were suspended and interrupted 'by a concourse of patients.' Notwithstanding this complaint, however, it appears that he found leisure for the composition of some of his most celebrated political productions." P. 114.

These followed each other in rapid succession.

"The following are the names and dates of these writings: A Word in Defence of the Bill of Rights against Gagging Bills. Where would be the Harm of a speedy Peace? Both published in the Winter of 1795: An Essay on the Public Merits of Mr. Pitt: A Letter to Mr. Pitt on the Scarcity; both in 1796: Alternatives compared, or, What shall the Rich do to be Safe? 1797."

These tracts are written with much spirit, energy, and manly freedom: the argumentative parts are occasionally enlivened by the warm colouring of a powerful imagination; and sometimes an admirable vein of irony is introduced. We regret that our limits, and the nature of this Journal, preclude us from inserting some of the numerous extracts which Dr. Stock has cited from these productions, which, independently of their literary excellence, do great honor to Dr. Beddoes, as a man of humanity, keen penetration, and inflexible integrity.

Dr.

Dr. Beddoes' opinion of the zoonomia which appeared about this time, and of which he had seen the proof sheets, merits a particular notice. Of the first volume he observed in a letter to a friend:

"Dr. Darwin has in the press a work of the most astonishing ingenuity, and I believe I may add, truth. It is a treatise upon the laws of animal nature, which he has been the first to discover fully. It is, as to materials and arrangement, perhaps the most original work ever composed by mortal man."

Of the second volume he remarked, "Dr. Darwin's second volume is out, and his analysis of morbid phenomena is one of the greatest exertions of the human understanding. The foundation of the most important and difficult science is fairly laid. There will be an end of medical imposture in due time; and if the profession continue to exist, the number of its members will be wonderfully diminished."

Towards the end of 1796, Dr. B. published the fourth and fifth parts of the Considerations on Factitious Airs.

"The fourth part consists of reports of cases treated by the pneumatic remedies. The fifth contains a variety of miscellaneous articles. The greater part of it is occupied by a description of Mr. Watt's implified pneumatic apparatus, drawn up by the inventor; and including many hints and cautions with respect to the preparation or employment of the gasses."

In 1797, he published, "Suggestions towards setting on foot the projected establishment for ascertaining the powers of factitious airs in medicine.."

He also wrote a small volume of reports of cases in which nitrous acid had been administered, and an introductory lecture of a course of lectures on anatomy, which his biographer ranks amongst the most valuable of his publications.

The following year, Dr. Beddoes had the satisfaction of opening the pneumatic institution, and of obtaining the assistance in superintending it of a gentleman, whose rising talents Dr. Stock observes—

"Eminently qualified him to enter into his views, and to assist in ascertaining and developing the resources of pneumatic medicine; and the more mature expansion of whose powers has since at once established his own fame, and extended the scientific reputation of his country. In one of the most remote parts of Cornwall, a young man, only nineteen years of age, 'with little access to philosophical books, and none at all to philosophical men,' during the course of an education, designed only to qualify him to act as a country practitioner of medicine, detected some inconsequent reasoning upon Caloric, which deformed the French theory of chemistry, struck out new views both upon that subject and upon lights, and supported them by a variety of novel experiments, ingeniously conceived and diversified." P. 154.

After this extract we need scarcely mention that this gentleman was Mr. Davy, who now occupies a foremost station in the ranks of science.

*(To be continued.)*

*Practical Observations on morbid Enlargements of the Testicle, &c.* By THOMAS RAMSDEN, Esq.

(Continued from page 177.)

IN the preceding number we endeavoured to lay before our readers a general statement of the principles of this work, which, both from the character of its author, and from the novelty of remark it promises, cannot fail to interest that particular department of the medical profession, to which it peculiarly applies. We now purpose to enter on an analysis of its contents.

The work opens with an assertion that many of the diseases of the testicle depend on a *latent* irritation within the urethra. This species, form, or degree of irritation, is denominated latent, on account of its extreme subtlety, and because the change in the membrane of the urethra from a natural healthy state is of a concealed nature, is shewn only by effects on other parts, and passes unobserved by the patient.

"The power of this principle to effect a derangement of the testicle can be exemplified in the gland itself; and may also be inferred from analogy in a variety of instances, in which other parts of the body (much less susceptible than the testicle, and situated also at a greater distance from the source of excitement than that gland is from the urethra) are occasionally placed under induration, from causes equally subtle and unobserved as that which is so frequently concealed within the urinary passage."

This subtle or insensible irritation is considered to constitute the basis of many formidable complaints, and is a fertile source of morbid derangements.

"Many instances of the baneful effect of this latent yet powerful agent of disease daily present themselves in various parts of the system, but which we have not been accustomed to trace to this particular source; I shall therefore introduce a few familiar examples of the general influence of insensible and slight irritation, previous to the more particular examination of its effect on that important gland, which is exclusively the subject of the present enquiry.

"When a susceptible or bloodshot point of membrane exists within the cavity of a joint, although so slight and subtle as to create no sensible uneasiness to the patient, it will cause such enlargement and induration of the adjoining parts as has been frequently mistaken for an expansion of the condyles themselves; this state of parts commonly *

* I believe I might say always, instead of commonly. I am indeed of opinion that a bloodshot state of membrane is the common basis of the white swelling; in other words, that the chronic enlargement and induration of parts surrounding joints, and the hectic fever which ensues, are solely dependent on such a source of slight excitement within their cavities. I ground this opinion on the strict analogy which the progress of white swelling seems to exists in that derangement of the knee joint which is called the white swelling.

"A similar effect takes place in those affections which we term scrofulous, and which seem to commence in the medullary parts of bones, in the tibia for instance, when a partial absorption of the body of the bone is taking place, or any very minute particle of osseous matter is slowly making its way towards the surface, the surrounding parts, without being in any degree inflamed or painful, will increase in bulk, and assume a resistent hardness, bearing every external resemblance to an increase of bony substance: yet these appearances immediately subside on the exfoliating bony particle being set at liberty.

"The common corn on the toe or foot is capable of inducing a flinty induration and enlargement of the inguinal glands, without in itself being sufficiently painful to awaken the attention of the patient to the part on which it is situated; in these cases, indeed, not only the source of irritation, but the affected gland also, will be so entirely free from uneasiness, that the induration will often advance to a considerable size before the patient becomes conscious of it.

"The mischiefs of insensible irritation frequently extend beyond those parts which are first exposed to its influence; when, for instance, it has occasioned an induration of any distant or neighbouring gland, such gland in its turn becomes a source of excitement to others, until the whole lymphatic system successively be affected.

"Latent irritation, besides inducing enlargement and hardness of glands, and other parts as rugged and resistent as that state which we frequently call scirrhus, has the further property of placing distant or neighbouring parts under characters and symptoms which are generally believed to be distinctly characteristic of certain constitutional affections.

"Thus in cases of confirmed syphilis, long after the venereal virus has been eradicated by a sufficient and judicious use of mercury, and the patient has been apparently restored to health, ulcerations will take place in the throat or on the palate, so exactly resembling the symptoms of venereal disease in these parts as to deceive even an experienced practitioner; yet such ulcerations eventually disappear on the exfoliation of some particle or portion of bone from the interior of the nose, which had latently produced the mischief.

"A singular example of the effect of subtle and distant excitement is afforded in worm cases, in which the sympathetic irritation within the nose, by long continuance, will cause thickening and abrasion of the membrane, and even exfoliation of the bones. These appearances in children have been occasionally mistaken for hereditary venereal disease; and a hasty, incautious expression of such an opinion by a professional man has sometimes involved parents in the deepest distress."

Two cases of *steatoma*, rendered malignant by common irritation, are given in exemplification of the principle Mr. Ramsden labours to establish.

---

to bear to those morbid derangements of various parts of the system which can be traced to similar sources of latent irritation.

From

From these analogies, the main object of the volume, diseases of the testicle, is entered upon. It appears that enlargement and induration of the testicle often arises from stricture in the urethra, not known to have existence; and a strongly marked case is given, where the disease resisted every remedy, but was at length removed by the bougie. In this case it was not probable that the stricture had been the result of gonorrhœal inflammation, but arose from some other irritation.

"The causes tending to produce a derangement of the membrane of the urethra are too numerous to admit of being distinctly specified; many of them are very remote, and others probably too minute and latent to be discovered by surgical investigation. In general it may be said, that whatever occasions a frequency of muscular action upon the urethra, or a frequency of excitement within it, or whatever induces a temporary inflammation of the canal, may establish a state of irritation in its membrane.

"Thus constitutional irritability, high living, excess in venery, indulgence in onanism, gonorrhœal inflammation, irritating injections, calculi in the bladder or kidnies, piles, and other affections of the rectum, &c. may become, through the medium of increased action, excitement, or inflammation within the urethra, the remote causes of morbid derangement of the testicle."

There is a state of the urethra, or unnatural diminution of its external opening, upon which Mr. Ramsden more particularly dwells, as being a fertile source of derangement in the membrane which lines that canal.

"In the majority of persons who apply for assistance under complaints of the testicle or of the urethra, which are not to be traced to gonorrhœal inflammation, the urethra will be found to be more contracted at its orifice than at any other part of the canal. This state of its extremity is very different from the stricture which is sometimes situated immediately within the aperture, as well as from that appearance of this part, which is called the blind urethra.

"This diminution of the orifice of the urethra is occasioned by membranous fence, which partially closes up the lips of the extremity of the canal; it may sometimes be an original mal-formation; yet I apprehend it is for the most part produced by cohesion during infancy, and is analogous to the union we sometimes see between the prepuce and glans penis in the male child, and between the nymphæ in the female

"Unimportant as this diminution of the extremity of the urethra may at first appear, many valuable facts may be deduced from it. It is the only instance, I believe, in which an immediate cause of spasmodic stricture in that canal can be demonstrated, by occasioning too strong and too frequent action of the adjacent muscles upon its diameter; at the same time, it affords an excellent illustration of the manner in which other causes, although more remote, may either predispose the urethra to stricture, or establish in its membrane a state of latent irritation.

"Whenever this membranous fence exists, or whenever the orifice of the urethra, from any cause, is smaller in its diameter than that of other parts of the canal, a sudden check is opposed to the free exit of the urine or semen at each attempt to propel them, and the increased muscular action, which is induced by the revulsion of those fluids upon the membranous part of the urethra, becomes at length the foundation of some of its diseases.

"Patients who have not been aware of this peculiarity of structure at the extremity of the urethra, have frequently, on my pointing it out to them, described to me the painful check and sense of distension they have experienced at each act of passing urine or semen; but for which they could never before satisfactorily account.

"The presence of this membranous fence, besides being the cause of derangement in the membrane of the urethra, also becomes the means of retarding its recovery, after disease has been there established: it aggravates all the symptoms in gonorrhœa, and if stricture has taken place, the removal of that state of membrane will be interrupted by the difficulty of introducing a bougie of sufficient size to make the necessary pressure on that part where the stricture is situated."

If we understand Mr. Ramsden aright, this mal-formation of the extremity of the urethra, to which he gives the odd term MEMBRANOUS FENCE, is not the immediate but the remote cause of indurated testicle. It produces that unobserved morbid state of urethra, here called *latent irritation*, from which arises a gradual, and for a considerable time, unnoticed enlargement of the gland. As this morbid state of the canal of the urethra, arising from the *membranous fence*, is unknown to the patient, and, according to Mr. Ramsden's statement, hitherto, as well as the membranous fence itself, unobserved by the surgeon, it will require a peculiar dexterity in the management of the bougie, and an extraordinary portion of delicacy in the tactus, however erudite on other occasions, to ascertain its situation. On this subject the following instructions are given.

"Latent irritation may be established at any part of the urethra between the bulb and the bladder. When this is the consequence of previous inflammation, it is confined to some distinct and acutely sensible point of the membrane, which bleeds on the slightest pressure of the bougie.

"When it is the result of preternatural muscular action, or of excitement, it is not confined to so distinct a point, neither is it so acutely sensible; but consists rather of a tenderness of the membrane, and particularly of that part of the canal which is within the prostate gland; it will also bear the pressure of the bougie without either bleeding at all, or not to that degree as in the former instance.

"As the state of membrane which constitutes a source of derangement to the testicle is only discoverable by the bougie, and the immediate object of the enquiry is to detect an unhealthy state of urethra not indicated by obstruction, and sometimes confined to a minute point, the greatest greatest gentleness and accuracy will be requisite in the examination.

"The bougie should be larger at one extremity than at the other, and should be of sufficient size at its larger end to occupy the whole diameter of the canal without distressing it by distension: previous to its being used the bougie should be oiled, and drawn through the fingers until it has acquired a proper degree of curvature to correspond with the sweep of the urethra: if a bougie be introduced which does not fill up the area of the canal, it will not only be liable to entangle in the lacunæ and thus embarrass the enquiry, but will miss or pass over any obscure point of irritation, upon which some degree of pressure ought to be made. A bougie also which has not been previously tempered and curved will stop at the sweep of the canal, and will not pass forward into the bladder, until sufficient force has been applied to make it, by pressure against the membrane, adapt itself to the rising course of the passage. Such a degree of pressure will produce some hesitation and a sense of pain in the most healthy urethra, which may lead an inexperienced practitioner to suppose he has detected a stricture or unhealthy state of membrane, when in reality no such state exists.

"The bougie, prepared as above described, is to be gently and gradually introduced with its larger extremity foremost: when it reaches the seat of irritation, which I have before said will be found between the bulb and the bladder, if such seat be confined to a distinct point of the membrane, the patient will express an acute pricking sensation, and the bougie on being withdrawn will generally be followed by some drops of blood. But when the seat of irritation consists in a derangement of a greater extent of membrane, the patient will describe a sensation of tenderness or soreness rather than of acute pricking, and the removal of the instrument will be seldom followed with any appearance of blood.

"In each of these deranged states of the canal, more or less of spasm will occasionally occur during the act of passing the bougie; if the unhealthy state of membrane is limited to a distinct point, the spasm will be momentary, and the bougie will be felt as if suddenly to start from the affected part; but where a greater extent of membrane is deranged, the spasm will be of longer duration, and the extremity of the bougie will appear to be gently grasped during the remainder of its passage into the bladder."

When an indurated enlargement of the testicle exists with either of the above states of the urethra; that is, with a *latent irritation*, either arising from previous inflammation, from preternatural muscular action, or from excitement, Mr. Ramsden assures us that his experience authorizes him to say, that such derangement of the gland is to be considered as dependent upon the altered state of the urethra; and that the surgeon must direct his attention to the restoration of the natural state of that canal, in order to relieve the affection of the testicle.

To this elucidation of the causes of disease in the testicle, investigations of particular *morbid enlargements of that gland*

*gland* naturally follow.* These investigations begin with SCLEROCELE, *or indurated testicle from latent irritation within the urethra; and its morbid characters and progress.*

"The alteration which latent irritation first produces in the testicle, for the most part consists in an enlargement and induration of the epididymis, very much resembling that state in which the epididymis is frequently left after hernia humoralis. Sometimes, however, the body of the gland is the first part which becomes hardened, at others the induration will commence in the spermatic chord. As the induration advances it acquires a peculiar callosity and cragginess; and the vas deferens, the epididymis, the body of the testicle, and the spermatic chord, all partaking of the derangement, eventually become blended in one hardened, irregular mass, in no way, that I am aware of, differing in outward character, or immediately distinguishable, from that morbid alteration of these parts which has been generally denominated scirrhus.

"During the progress of such morbid alteration in the gland, an undue effusion of serous fluid will occasionally take place within the tunica vaginalis, or underneath the coverings of the spermatic chord; in the former case producing a hydro-sclerocele, and in the latter a hydro-spermato-sclerocele.† When a testicle has reached this state of disease, the progress of its farther derangement becomes uncertain and indefinite, but the most usual course is a languid suppurative inflammation at the lower part of the scrotum, with the projection of an irregular granulating fungus through the aperture of the abscess.

"Notwithstanding the sclerocele will in some few instances, on account of the constriction of fluid, become painful at a very early period of the induration, yet in most cases the morbid derangement is so subtle at its commencement, and its progress is so extremely gradual, that the disease seldom becomes the object of surgical care, until the patient's

---

* It is an observation of practical importance, that "when a testicle from any cause whatever becomes deranged, the true pathognomonic characters will be obscured or lost as soon as the painful or inflammatory state of such derangement commences: in other words, the sclerocele, the scirrhus, the sarcocele, the scrophulous testicle, and that which has been called venereal, though characterized by certain distinct features previous and even up to the time when they become painful, or are rendered so by accident, will from that period all assume a common mixed character, so alike in appearance, that unless a surgeon attends carefully to the preceding history of the disease, and to the symptoms which have marked the progress of such derangement, he will not be able to discriminate these diseases in their more advanced stages." "The features which distinguish the sclerocele, the scirrhus, and the sarcocele from each other, will be found to be less strongly marked between the sclerocele and scirrhus, than between either of those two affections and the sarcocele. The state of the testicle, which has been called venereal, bears some slight resemblance to the sarcocele, but the scrofulous testicle differs from all."

† This is a consideration of great importance, since fluid, when tightly constricted, will oftentimes feel equally hard and resistent as the indurated gland or chord, and therefore add considerably to the obscurity of the case on examination. Fluid, when tightly bound down upon an irritable testicle, is also capable of producing general symptoms in the system which might seriously mislead an inexperienced practitioner.

attention attention is either attracted by the inconvenient bulk of the tumor, or some accidental circumstance occurs, and diverts it from the usual course. There is scarcely an instance of sclerocele of the testicle which does not corroborate this remark, by bearing incontestible evidences of its having existed long before the time at which the patient dates the discovery of his complaint. On this account a patient will frequently attribute the enlargement of the gland to hard riding, to a strain, to a cold, to a fever, or to some other occurrence which has had no influence in the production of the complaint, but merely induced disturbance in a testicle, previously indurated, and ready to become enlarged upon the application of any exciting cause.

"We sometimes however meet with patients whose cases are to be considered as an exception to this general rule or course, in whom the common sclerocele of the testicle will advance with great rapidity, independently of any constricted fluid, and of all the accidental circumstances to which I have referred. These are persons who are constitutionally irritable, and in whom the testicle and membrane of the urethra partake of the general susceptibility of the system.

"But if a surgeon be consulted in a case of sclerocele of the testicle before the gland has taken on a diseased action of its own, the rapidity with which such derangement has proceeded will be found to be rather favourable to the cure than otherwise.

"I have observed in cases in which the seat of irritation within the urethra is acutely sensible, and in which the enlargement of the testicle has been rapid, that the cure is much more obedient to the treatment by the bougie than in those in which the source of irritation is less sensible, and in which the gland has been more slowly habituated to its influence. In the former cases, also, the removal of the source of irritation will be always sufficient, alone, to perfect the recovery of the testicle; but in the latter it will frequently be requisite after the cause has been removed from the urethra, to have recourse to local mercurial friction for the purpose of dispersing some remaining point of induration in the gland.

"The preceding observation, however, is to be considered as only applicable to that rapidity of progress in the sclerocele of the testicle which is solely dependent upon an increased degree of susceptibility in the seat of irritation within the urethra, and must on no account be confounded with the quickened course of disease, which is occasionally excited by general indisposition, or by injury to the gland itself. These cases differ essentially from each other, and require a very different mode of practice. In the former the early treatment of the urethra will stay the progress of derangement in the testicle, and probably effect a speedy cure; but in the latter such resort to the immediate use of the bougie will frequently be injurious, and by aggravating all the symptoms, may place the gland beyond remedy.

"If we reflect on the anatomy of the testicle, we must believe that when the breaking open or the actual exposure of its organic structure takes place, the functions of the gland will be destroyed; and this state of parts, which has been commonly called the "spoiling of the testicle," has at all times been admitted as a sufficient ground for advising the operation of castration. It must be of great importance, therefore, to ascertain whether we are not liable to be misled by appearances, and to to condemn a testicle to the knife, under the idea of its being spoiled, even whilst its vascular structure yet remains entire.

Of the several morbid affections of the testicle, which are presumed to be fatal to its vascular structure, the formation of matter within the body of the gland may be considered as the most frequent. I suspect, however, in many instances in which abscess takes place within an indurated enlarged testicle, that the suppurative process is restricted to the previously thickened cellular substance, which by its intervention must necessarily be supposed to maintain the vascular structure of the body of the gland, as well as the continuous convolutions of the epididymis, in a state of extensive separation*.

"When, in consequence of suppuration within the sclerocele, a fungus is projected from the lower part of the scrotum (which is a very common occurrence), such morbid affection has been extensively admitted as authorizing the extirpation of the testicle, and I will acknowledge I have myself, on some occasions, acted upon such opinion; but later observation has shewn even this state of disease, in the early period of such fungated appearance, to be frequently confined to the cellular substance, or to the coats of the gland, and for the most part therefore totally independant of the vascular structure.

"If in true sclerocele the organic structure was liable to be "spoiled" at all times when suppuration takes place within the substance of the tumor, we should surely obtain frequent proof of such a fact, and yet I can truly declare (in cases in which I have had an opportunity of controling the derangement in the gland by the timely and early treatment of the urethra), I have scarcely seen a single instance where I had reason to suppose the functions of the gland to be eventually "spoiled," merely in consequence of matter having formed amidst the induration.

"Allowing, however, the exposure of the organic structure of the indurated testicle to be sometimes an immediate consequence of suppuration within its substance, when such an effect results solely from a source of irritation within the urethra, it would by no means justify the early removal of the part as a measure of necessity.

"Even under such distressful circumstances, if the patient be content to retain the gland in its defective state, the treatment of the urethra will, I believe, in most instances, prove competent to the preservation of the remaining portion, and render it in future perfectly inoffensive to the constitution; provided such treatment be resorted to, when the patient is not suffering under general indisposition, and before the neighbouring parts have taken on a diseased action in consequence of the long continuance of irritation.†

---

* It may be observed in the common varicocele of the testicle how easily its organic structure admits of extensive separation.

† This is one of the circumstances which distinguishes the sclerocele essentially from the sarcocele. When the structure of the testicle is exposed by that suppurative process, which is merely a consequence of irritation within the urethra, the gland may be restored to a quiescent state, and may be so far recoverable; but when the organic structure of the testicle is exposed by the progress of sarcocele, such a compromise must not be expected.

This observation applies also to the scrofulous testicle. Although large portions of the gland will slough away, in what is called the scrofulous testicle, yet in many instances the remaining parts will heal up, and continue through life without occasioning farther inconvenience.

These opinions are illustrated by the subsequent cases: "sclerocele of the testicle from latent irritation within the urethra; sclerocele of the testicle from latent irritation within the urethra, mistaken for omental hernia; sclerocele of the testicle in the groin; sclerocele of the testicle commencing within the body of the gland; sclerocele of both testicles, one of which suppurated, arising from latent irritation within the urethra; fungous sclerocele from latent irritation within the urethra, in which the induration commenced within the body of the gland. The next section treats of

SCIRRHUS *in the Testicle, and its morbid Distinctions.*

The difficulty of discriminating between sclerocele and scirrhus, is often a source of great embarassment to the surgeon, and of hazard to the patient. This is fully felt by our author, and he gives the following pathognomonic symptoms with doubt and hesitation.

"Whenever a testicle increases in size, is craggy on its surface, and of a stony hardness, whether with or without watery fluid within the tunica vaginalis, at whatever part of the gland the induration may have begun, the first enquiry ought to be whether such a diseased alteration be dependent upon an unhealthy or too susceptible a state of urethra. If in spite of a proper attention to the urethra, and of a general or local trial of mercury seasonably and judiciously used, such induration and cragginess continue to extend, and especially if severe darting pains* in the direction of the loins come on before the skin appears reddened and inflamed, the disease must be viewed as scirrhus."

A case of sclerocele is annexed, which had the characteristics of true scirrhus.

The testicle is occasionally met with in a state of considerable enlargement and induration, free from pain for the most part, without fluid in the tunica vaginalis, unattended with thickening of the spermatic chord; and though hard and resistent, yet retaining an uniform smoothness and equality of surface. This has generally been considered as having a syphilitic origin, and is here noticed in a section treating of that

*Morbid State of the Testicle which has been called Venereal.*

"This state of gland is unconnected with any derangement of the urethra; and being frequently observed to take place during the pro-

* Even this character should be received with caution and reserve. The common sclerocele, in an irritable habit, will be painful at an early period of the induration; and if accompanied with fluid constricted within an unyielding tunica vaginalis, will (in consequence of the pressure of such fluid against the gland) be attended with darting pains toward the loins.

gress of syphilis, and to yield to mercury, has been supposed to constitute one of the symptoms of that disease.

" In its general features it bears some resemblance to the sclerocele, the scirrhus, and the sarcocele; but in its particular characters it is essentially different from them all. It resembles the sclerocele and scirrhus in hardness and resistence, but differs from them in its uniform smoothness and equality of surface; it resembles the sarcocele in smoothness and equality of surface, but is distinguishable from it by hardness and resistance."

Mr. Ramsden doubts if this morbid state of the testicle ever has a venereal origin, but observes,

" Whether these opinions on the nature of that affection of the testicle which has been called venereal are well founded or erroneous, is perhaps of no great practical importance, since experience has taught us that whenever the gland presents the characters of that morbid enlargement, which I have defined, it will be proper to subject the patient to a judicious and well regulated course of mercurial frictions, which, for the most part, has the effect of restoring the gland to its natural state.

" This affection of testicle, however, is sometimes blended with the sclerocele; and whenever therefore a derangement of the urethra shall be ascertained to be present with it (I have already said that in every instance of morbid enlargement of the testicle the urethra should be examined), it will be right to resort to the occasional use of the bougie during the mercurial course.

" I have to remark also, that in a majority of instances where the case is of this mixed kind, the back part of the testicle will be found, on a careful examination, to be more rugged and unequal, than the general bulk of the tumor."

The next morbid alteration of the testicle, noticed by Mr. Ramsden, is,

### *The Scrofulous Testicle.*

" When a testicle increases in fullness of size, retaining in a great measure the outward features of the gland, is at the same time free from pain, or any affection of the spermatic chord, and is characterized by *a soft, pulpy, relaxed feel without elasticity;* such a state of the gland, is called a scrofulous testicle.

" Children and adults are equally liable to this affection. It occurs in those children who have light or red hair, a delicate skin, with a florid complexion, and such other general external appearances as are supposed to mark a disposition to scrofula, and especially in those who shew any tendency to mesenteric obstruction.

It takes place in those adults who are naturally of a weakly, delicate constitution, or whose system has been weakened, and general health impaired by a long residence in hot climates, or by an irregular course of life.

" In children it is no unusual occurrence to find, even before the testicle has acquired any considerable degree of fulness of size, the skin of the the scrotum to give way, the testicle repeatedly to slough, and the parts afterwards to cicatrize without much difficulty.

"But in adults the skin of the scrotum seldom gives way, so as to expose the body of the testicle until a very considerable increase of bulk* has taken place; yet such enlargement is not attended with pain or affection of the spermatic chord, neither is there any derangement of the health, farther than seems to arise from general debility.

"In such an enlarged state of gland the skin of the scrotum will redden and break sometimes in several places, and will expose different parts of the substance of the testicle in a state of slough. The progress of the sloughing, however, is commonly very slow, and only takes place at intervals; so that a patient will carry a diseased testicle of this description, from time to time throwing off sloughs and for many years, without the mischief extending up the chord, without much injury to the general health, and without being incapacitated for the common avocations of life.

"If the sloughing ceases before the gland be quite destroyed, the surface of the remaining portion frequently becomes elevated above the level of the skin of the retracted scrotum, and projects luxuriant granulations, which, from the difficulty of being restrained within proper bounds, very much impede cicatrization."

It is not contended that this morbid state of the testicle arises from irritation in the urethra; but that such irritation will induce upon a testicle previously disposed to, or suffering under scrofulous affection, such a change in its appearances as will materially alter its pathognomonic symptoms.

In the former part of this analysis, we observed that our author did not include *sarcocele* in his arrangement of morbid alterations in the testicle. He, however, described this disease in the prefatory part of his work, as differing specifically from *scirrhus*; that on dissection it displayed features peculiar to itself; that it was fleshy and elastic to the feel; was often found to contain within its substance partial collections of bloody sanies; and that it did not present the ligamentous radiated appearance, which in true scirrhus is uniformly observable. He also gives it a place in the body of the work; and we consider it to be an affection of such importance both to the patient and to the surgeon, inasmuch as it is intractable to medicine, and demands a prompt decision on the part of the practitioner, that we shall insert the whole of the section which treats on it.

* It is to be remarked, that when the scrofulous testicle is about to enter into its second stage, which is generally a short time before the skin begins to appear discoloured, it gradually loses its grand characteristic feature, viz. its soft, pulpy feel, and acquires a degree of general hardness very much resembling what is attendant on the first stage of that state of the testicle which has been called venereal.

" When the body of a testicle increases in bulk, feels fleshy and elastic, is perfectly smooth and uniform on its surface, without thickening of the spermatic chord, and occasions no pain or inconvenience but from its weight, or on being handled, I should suspect such affection of the gland to be sarcocele.*

" This disease is idiopathic, and has hitherto been incurable; although it may occasionally exist for years without making much progress, it is always liable to sudden and dangerous changes, which may place it even beyond the reach of an operation.

" As the disease proceeds, it induces derangement of the system, and the countenance of the patient assumes a peculiar sallowness of appearance.

" In the more advanced stage of sarcocele, even without the presence of pain, there will occur partial collections of fluid, commonly of bloody sanies, within the body of the gland, which present themselves by mamillary elevations of the tunica albuginea. The fluctuation in these elevations will be so distinctly felt as occasionally to mislead the surgeon, and induce him to puncture them, under a supposition that the bulk of the tumor wholly consists of a collection of fluid.†

" Such a mistake may lead to the most serious results, since it seldom happens that the gland will again subside into a quiet state.

" When the disease in its regular progress, or by the excitement of any accidental circumstances, reaches its painful or suppurative stage, it will lose its elasticity, assume the common characters of irritation, and projecting a painful gleeting‡ fungus from the ruptured part, extend with a rapidity which not only sets all means of relief from surgical treatment at defiance, but sometimes renders castration itself ineffectual.

" It will appear from the preceding observations that the fleshy elas-

---

* " If the body of the testicle, though enlarged and ' indurated' to some degree, be perfectly equal in its surface, void of pain, has no appearance of fluid in its tunica vaginalis, and produces very little uneasiness except what is occasioned by its mere weight, it is usually called a simple sarcocele, or indolent *scirrhus*." POTT.

† In some few cases of sarcocele a small quantity of fluid, but of a limpid kind, will also be found within the tunica vaginalis, and will be bound down so tightly on the diseased gland as to render it painful and progressive at an early period. I have a patient at this time under my observation with true sarcocele, and I have twice within the last eighteen months stopped the progress of the disease by cautiously evacuating a small quantity of fluid from the tunica vaginalis. I do not, however, venture to recommend the practice; and would gladly prevail upon my patient to part with his testicle, but he has hitherto resisted my advice, and will probably repent it, as the gland is daily liable to be placed beyond the reach of an operation.

‡ " Gleeting." This sort of discharge, or a discharge of " bloody sanies," is not a pathognomonic character, though it has hitherto been considered as such. When a fungus of any description is projected through the tunica vaginalis, the natural but excited secretion of that sacculus mixes with the purulent discharge of the fungus, and gives the " gleeting appearance." When this secretion of the sacculus happens also to be tinged with blood in passing over the irritable surface of the fungus, it constitutes "the bloody sanies," on which so much stress has been laid as a supposed character of cancer in the testicle.

ticity, ticity, with the total absence of induration* and cragginess, are the chief characters which distinguish sarcocele from other morbid enlargements of the testicle; on this account a collection of blood or watery fluid within a diseased or thickened tunica vaginalis may easily be mistaken for sarcocele.

"The fleshy elasticity of a testicle in the first stage of sarcocele is so similar to the feel of fluid through the medium of thickened membrane, that the most experienced surgeons have been deceived by it. This deception indeed is acknowledged to be so imposing, that it has now become an established point in practical surgery, invariably to puncture a supposed sarcocele, previous to the intended operation of castration.

"Since sarcocele is idiopathic, in all its stages incurable, and even in an apparent quiet state liable to sudden and dangerous changes, it will be the duty of the surgeon, as soon as he has distinctly ascertained its character, even before the gland has acquired a painful state, to explain to the patient the danger of his retaining a testicle under circumstances of such extreme hazard, and to submit to him the propriety of its removal."

The remainder of the volume appropriated to the diseases of this part of the frame, consisting of one hundred and forty pages, is occupied by observations on "*Watery Effusions into the Tunica Vaginalis.*"

(To be continued.)

* "Every species of sarcocele consists primarily in an enlargement, '*induration*,' and obstruction of the vascular part of the testicle." Vide Pott.

well

